# Characterising the Influence of Parasocial Experience on the Media Figures’ Audiences: A Scoping Review

**DOI:** 10.3390/bs16050808

**Published:** 2026-05-18

**Authors:** Luyang Li, Shiyu Wang, Kim Hua Tan

**Affiliations:** School of Media and Communication, Faculty of Social Sciences and Leisure Management, Taylor’s University, Subang Jaya 47500, Malaysia; lxm9306@gmail.com (L.L.); 0363604@sd.taylors.edu.my (S.W.)

**Keywords:** parasocial experience, parasocial interaction, parasocial relationship, parasocial attachment, social media, figures, audience

## Abstract

Parasocial experience describes the psychological, emotional, and behavioural bonds that audiences form with media figures—celebrities, fictional characters, and other on-screen personalities. It encompasses three related constructs: parasocial interaction, parasocial relationship, and parasocial attachment. Following the Joanna Briggs Institute framework and PRISMA-ScR guidelines, this scoping review synthesises 59 empirical studies published between 2019 and 2024. Results show that PSRs were the most studied dimension (*n* = 42), followed by PSI (*n* = 20). PSA, in contrast, appeared in just one study. Key characteristics across these studies include perceived familiarity, imagined intimacy, behavioural engagement, intuitive personality traits, and compensatory motivations. The review highlights PSA as a particularly underexplored yet affectively intense form of engagement. Its limited empirical attention reveals a critical theoretical gap and calls for further investigation into attachment-related audience behaviours.

## 1. Introduction

Contemporary media audiences no longer act as passive recipients. Instead, they engage with celebrities and other figures in more interactive and emotionally invested ways—participating in symbolic exchanges and developing perceived emotional bonds (Z. Zhang, 2023). This phenomenon—what scholars call parasocial experience (PSE)—captures the one-sided psychological bonds people form with media figures they have never met. Characterised by perceived intimacy, emotional reliance, and expressive behaviours, PSE has become central to media and communication studies (Tukachinsky & Stever, 2019).

Once dismissed as pathological (Groszman, 2020), PSE is now recognised as a common and complex feature of how audiences relate to media. It spans traditional celebrities (e.g., actors, singers, athletes) and newer entities such as social media influencers (SMIs) and virtual personas (Aw & Chuah, 2021; Zhou et al., 2024). As media becomes more personalised and participatory, parasocial bonds are embedded in everyday digital interactions.

Three constructs define PSE: parasocial interaction (PSI)—momentary perceptions of interaction during media use (Horton & Wohl, 1956); parasocial relationship (PSR)—enduring emotional bonds (Tukachinsky & Stever, 2019); and parasocial attachment (PSA)—deep affective ties marked by trust, longing, and dependence (Stever, 2013; Brooks, 2021).

Despite growing relevance, the literature remains fragmented. Many studies isolate constructs or behaviours, and PSA is often conflated with PSRs, limiting conceptual clarity.

This scoping review addresses these gaps by synthesising 59 empirical studies (2019–2024). Using the Joanna Briggs Institute methodology and PRISMA-ScR guidelines, it maps the conceptual contours of PSE, clarifies distinctions among constructs, and identifies key psychological and behavioural features.

### 1.1. Aims and Review Questions

PSE has gained increasing attention across cultural and academic contexts, yet existing studies remain fragmented, often focusing on individual constructs such as PSI or PSRs, with limited attention to PSA as a distinct category. To date, no comprehensive review has synthesised how different forms of PSE influence audience behaviours.

In addition, parasocial processes have been theoretically discussed as a form of mediated social contact, suggesting their potential to influence broader attitudinal and behavioural outcomes. This scoping review addresses that gap by systematically mapping empirical research published between 2019 and 2024. It aims to clarify the distinctions among PSI, PSRs, and PSA, and identify their key psychological and behavioural features. By doing so, it offers theoretical clarity and practical insights into how audiences engage with media figures, particularly in digital environments.

Following Mohamed Shaffril et al. (2021), this review is guided by the following questions:

Which aspects of PSE influence audience behaviours?
What are the main features of these influences?

These questions support a comprehensive and conceptually open synthesis of recent developments in the field.

### 1.2. Conceptual Distinction Between PSI, PSRs, and PSA

Despite the widespread use of PSE in media and communication research, its core constructs—PSI, PSRs, and PSA—are often used interchangeably, which blurs their distinct meanings. To clarify, this review distinguishes the three constructs along three dimensions: temporality, emotional intensity, and psychological function.

PSI captures the immediate, situational sense of interacting with a media figure while watching, listening, or scrolling. It is usually short-lived and episodic, kicking in when audiences feel directly addressed, sense reciprocity, or experience a feeling of co-presence (Horton & Wohl, 1956; Schramm & Hartmann, 2008). PSI primarily operates at the level of cognitive and affective engagement, shaping how audiences attend to, interpret, and emotionally respond to media figures in real time.

In contrast, PSRs represent a more enduring and stable form of connection. They develop over repeated instances of PSI and reflect ongoing, one-sided relationships that audiences perceive as similar to friendship or companionship (Tukachinsky & Stever, 2019). PSRs are characterised by emotional continuity, perceived familiarity, and relational commitment. Unlike PSI, which is moment-bound, PSRs persist beyond specific media encounters and become integrated into the audience’s social and emotional world (e.g., Z. Zhang, 2023).

PSA, by comparison, constitutes the most affectively intense and psychologically internalised form of parasocial experience. Rooted in attachment theory (Bowlby, 1982; Stever, 2013), PSA involves deeper emotional bonds marked by trust, longing, and a sense of emotional security (Tukachinsky & Stever, 2019). While PSRs reflect relational continuity, PSA reflects attachment-based dependency, where the media figure may function as a symbolic attachment figure (e.g., Scherer et al., 2022). This distinction is critical, as PSA operates not merely as a relationship but as an internalised emotional structure that can influence identity, regulation of affect, and perceived psychological support.

Taken together, these three constructs can be understood as forming a continuum of parasocial engagement: from momentary interaction (PSI), to sustained relational bonds (PSR), to deep attachment-based connections (PSA). Clarifying these distinctions is essential for advancing theoretical precision and avoiding conceptual overlap in the study of parasocial processes.

## 2. Method

In this study, we used Arksey and O’Malley’s (2005) methodology as an overall guiding approach to analyse, synthesise, and characterise the influence of PSE on the audience. The Joanna Briggs Institute’s methodological framework was used for a more structured and systematic approach to the selection and charting of data by adopting the PRISMA-Scr and Inclusion and Exclusion Criteria. This is to ensure reproducibility of the Search Strategy.

### 2.1. Defining and Measuring PSE

This review categorises PSE into three constructs—PSI, PSRs, and PSA—and uses them as the framework for classifying the studies. Given the historical overlap between these concepts, we defined each one clearly for this review and identified available measurement tools wherever possible.

PSI refers to short-term, momentary perceptions of connection during media exposure. Studies were classified as PSI-focused when they examined responses such as feeling addressed, emotional uplift, or symbolic participation (e.g., commenting, following). These experiences span cognitive, affective, and behavioural dimensions. Common instruments include the Parasocial Interaction Scale (Schramm & Hartmann, 2008) and the Experience of Parasocial Interaction (EPSI) Scale (Hartmann & Goldhoorn, 2011), which assess intimacy, perceived presence, and reciprocity (see Table 1 and Table 2).

PSRs denote sustained, emotionally grounded connections resembling one-sided friendships with media figures. Two audience-level traits commonly underlie PSRs: (1) intuitive personality types—individuals predisposed to forming long-term emotional bonds; and (2) compensatory needs—audiences seeking to fill emotional voids through mediated connections. While PSRs lack a standardised measurement scale, it is often conceptualised as a cumulative outcome of repeated PSI, with longer-term engagement inferred through items measuring commitment or loyalty.

PSA, the most affectively intense form of PSE, describes deep emotional bonds characterised by longing, emotional security, or identification. Rooted in attachment theory (e.g., Bowlby, 1982; Stever, 2013), PSA reflects stronger internalised functions such as trust and symbolic proximity. Due to the absence of dedicated quantitative tools, most studies employ qualitative approaches or adapted measures to capture the depth of attachment and psychological dependency. This signals an urgent need for PSA-specific instruments, especially within fan studies and audience psychology.

In cases where studies addressed more than one construct, classification was based on the dominant theoretical focus and methodological emphasis.

### 2.2. Search Strategy

This review followed Arksey and O’Malley’s (2005) five-stage scoping review framework and was guided by the PRISMA-ScR guidelines and the Joanna Briggs Institute’s inclusion criteria (Peters et al., 2020).

Searches were conducted across ScienceDirect, Scopus, and Google Scholar using Boolean operators (‘AND’/‘OR’) and keywords such as ‘parasocial experience audience’, ‘parasocial interaction audience’, and ‘parasocial audience behaviours’. Reference management and duplicate removal were handled via Zotero (Bai & Tan, 2024). 

From 745 initial results, 567 duplicates were removed. Titles and abstracts of the remaining 178 records were screened based on the following criteria:

- Peer-reviewed and open-access;
- Published between 2019 and 2024;
- Focused on PSI, PSR, or PSA and their impact on audience behaviours.

**Figure 1:**
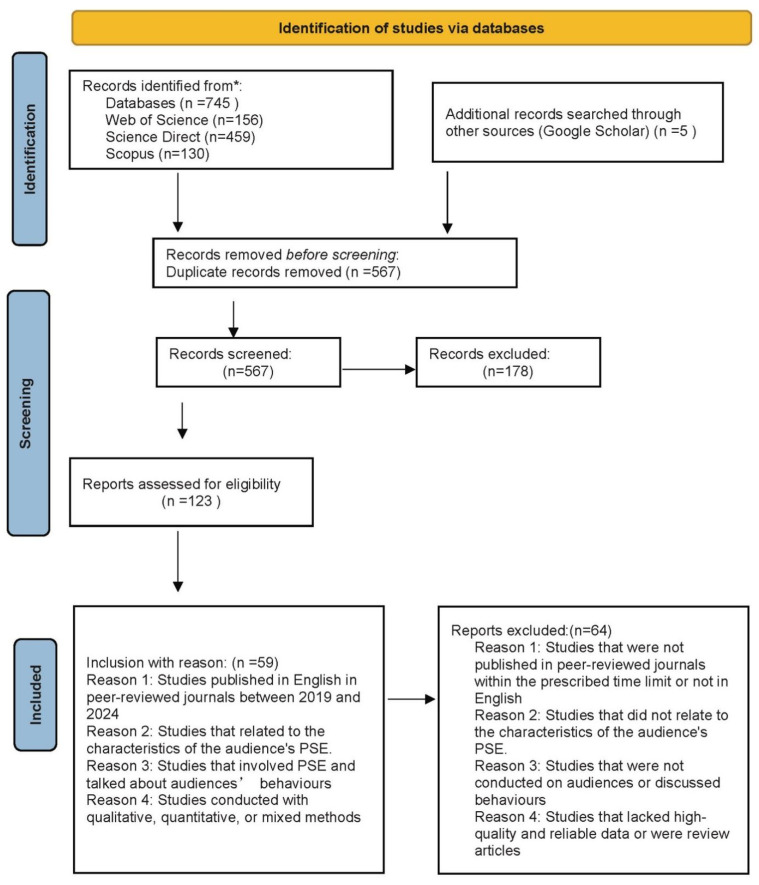
This figure is adapted from the PRISMA 2020 flow diagram (Page et al., 2021) and revised to reflect that only database searches were used for study identification. The asterisk is part of the original PRISMA template and does not carry additional meaning in this figure.

After full-text screening, 123 studies were excluded for lack of relevance. In addition, five studies were identified through manual searches, resulting in a final sample of 59 studies. The detailed selection process is illustrated in Figure 1. Details are provided in Table 3. To ensure comprehensiveness, backward and forward reference searching was also conducted, resulting in five additional studies. The restriction to open-access studies was applied to ensure accessibility and reproducibility of the review process. However, this decision may have excluded relevant studies published behind paywalls.

## 3. Results

This section presents the descriptive findings from the 59 studies included in our review. We organised the findings to map out the research landscape from 2019 to 2024, looking at publication trends, geographic spread, the kinds of media figures studied, methods, samples, and which dimensions of PSE got the most attention. These descriptive patterns lay the foundation for the interpretive discussion that follows.

### 3.1. Publication Year

Between 2019 and 2024, a total of 59 peer-reviewed empirical studies were included in this review. The year 2020 marked 11 studies (18.6%), followed by 2021 with 12 studies (20.3%) and 2022, which contributed 14 studies (23.7%), representing the highest publication output. The year 2024, though still ongoing at the time of this review, had already produced eight studies (13.6%), indicating sustained academic interest. The lowest number of studies were published in both 2019 and 2023, each accounting for seven publications (11.9%).

These figures reflect a notable rise in scholarly attention to parasocial experience beginning in 2020, coinciding with the global acceleration of digital media use during the COVID-19 pandemic. This upward trend continued across the subsequent years, suggesting that parasocial behaviour has become an increasingly significant focus within media and communication research.

### 3.2. Type of Media Figures

A clear imbalance was found in the types of media figures studied. Social media influencers (SMIs) dominated, appearing in 26 studies (44.1%), followed by singers (13.6%), television characters (10.2%), actors (6.8%), and idols (5.1%). Less common were athletes, movie characters, reality TV personalities, politicians, and socialists—each featured in one study (1.7%). One study explored audience connections with a generative AI chatbot, indicating growing interest in virtual entities. Five studies (8.5%) did not specify a media figure. The prominence of SMIs reflects a shift toward more accessible, interactive, and algorithm-driven celebrity forms, likely intensified by the COVID-19 pandemic.

### 3.3. Type of PSE Categories

Based on the 59 included studies, PSE was categorised into PSI (33.9%), PSR (69.5%), and PSA (1.7%). Among PSI studies, 11.7% focused on cognitive, 16.7% on affective, and 5.0% on behavioural responses. PSR studies were primarily driven by compensation needs (39.0%) and personality traits (26.7%). Only one study (1.7%) addressed PSA, highlighting deep attachment grounded in emotional reliance.

## 4. Discussion

Classic definitions go back to Horton and Wohl (1956): PSI as a “simulacrum of conversational give-and-take,” and PSR as a “seeming face-to-face relationship between spectator and performer.” (Horton & Wohl, 1956; Tukachinsky & Sangalang, 2016). But despite their age, these terms have been used inconsistently, causing confusion in the field. So over the last ten years or so, researchers have pushed for clearer definitions—not just to tell PSI, PSR, and PSA apart, but also to see how they are connected (Schramm & Hartmann, 2008; Hartmann & Goldhoorn, 2011; Tukachinsky & Stever, 2019). Across the 59 studies we reviewed, a clear pattern emerged: different dimensions of parasocial experience seem to operate through distinct mechanisms. PSI tends to shape viewers’ immediate cognitive, emotional, and behavioural responses during media use. PSRs, by contrast, link more closely to lasting patterns like personality traits and the need for emotional compensation. PSA, though rarely studied, points to deeper, attachment-based dynamics—emotional dependence, longing, and a sense of psychological security. The following sections will specifically examine how these characteristics impact audiences and how these effects manifest. Taken together, these findings paint a picture of PSE as a multi-layered process, playing out across interactional, relational, and attachment-based levels. PSI captures immediate engagement, PSRs reflect sustained relational bonds, and PSA represents deeper attachment dynamics. Integrating these dimensions provides a more comprehensive framework for understanding how audiences connect with media figures, not only as sources of entertainment, but as meaningful social agents within mediated environments.

While the descriptive results provide an overview of the research landscape, several important patterns emerge. First, the dominance of PSRs in the literature suggests a strong emphasis on enduring relational bonds over momentary interactions, indicating a conceptual preference for stability and continuity in audience–media connections. Second, the prevalence of social media influencers reflects a broader shift toward algorithmically mediated intimacy, where frequent interaction and self-disclosure enhance perceived closeness. Third, the near absence of PSA highlights a critical gap in capturing deeper emotional dependency, suggesting that current research may underestimate the intensity of audience–media bonds.

### 4.1. Parasocial Relationship

#### 4.1.1. Personality Type

PSRs often emerge when audiences’ self-awareness merges with the symbolic persona of a media figure—what Tukachinsky and Stever (2019) call the “integration stage.” A key psychological trait driving this process is intuitive personality, defined by imagination, emotional openness, and fantasy orientation (Myers & Myers, 2010). This trait supports long-term, emotionally stable PSRs.

Seventeen studies in this review identified intuitive personality as a key factor in PSR development, especially among audiences who interpret media figures as emotionally resonant companions (e.g., A. Yang & Shim, 2020; Deng et al., 2022). Often referred to as “dreamers,” these individuals project internal emotions onto media figures, fostering sustained emotional loyalty (Tukachinsky & Stever, 2019). Eight studies focused specifically on fans, who seem especially prone to deep PSRs. Most of the relevant studies (10 out of 17) looked at singers, actors, or social media influencers (SMIs)—figures who build intimacy through personal narratives and frequent interactions (e.g., Sharon & John, 2024). Intuitive personalities also show up in behaviours like emotional advocacy and purchases—actions that reflect lasting psychological commitment and a sense of identity alignment.

#### 4.1.2. Compensation Needs

Compensation works differently. It is a context-driven mechanism that stems from real-life loneliness or a lack of emotional connection (Wang et al., 2018; Stein et al., 2024). PSR offer temporary emotional support, especially during difficult times, but they tend to fade once real-life needs are met. In that sense, compensation extends real relationships rather than replacing them (Tukachinsky & Stever, 2019; Groszman, 2020).

Among the 42 PSR-related studies reviewed, 25 examined compensation. These studies show that audiences form emotional bonds with media figures they see as friends (W. Gong & Li, 2019) or even as “parakin”—a kind of extended family relation (Z. Zhang, 2023)—especially when figures share personal content (e.g., Pope & Rose, 2024). While intuitive personality fosters long-term loyalty, compensation is a short-term coping strategy. These processes do not just meet emotional needs—they can also lead to broader social outcomes, like reducing prejudice (Bond, 2021), shifting attitudes toward social groups, or shaping how people morally evaluate media figures (You et al., 2023). Compensation appears more broadly across user types, particularly in social media contexts, where 12 of 25 studies focused on SMIs. Unlike intuitive personality, compensation cuts across diverse groups, including adolescents, fans, and LGBTQ individuals. This may explain why SMIs have become such effective sources of emotional support.

### 4.2. Parasocial Interaction

#### 4.2.1. Cognitive

Cognitive engagement plays a key role in shaping PSE by influencing how audiences process and relate to media figures (Schramm & Hartmann, 2008; Tukachinsky & Stever, 2019). One central feature is the sense of really knowing the figure. When media figures share personal experiences or come across as relatable—especially on social media—audiences feel a stronger sense of familiarity, which can shape their self-concept and identity. Another cognitive trait is being less critical in the event of scandals. Fans, in particular, tend to idealise media figures, attributing positive traits to them. As a result, they are often more forgiving when transgressions occur, although severe misconduct can still lead to a rupture in the PSE.

Only five studies in our review looked at cognitive PSI. Most of them highlight its role in building perceived familiarity, especially with SMIs who engage audiences through frequent, personalised content (e.g., A. L. Zhang & Lu, 2022). This kind of cognitive resonance builds trust and deepens the sense of emotional closeness.

#### 4.2.2. Affective

Affective responses are the emotional reactions—positive or negative—that audiences have toward media figures (Tukachinsky & Stever, 2019). One key affective pattern is that the media figure simply makes people feel better—lifting mood and reducing bias, for instance by fostering empathy for marginalised groups. Another central affective feature is a feeling of intimacy. This perceived closeness fuels emotional investment and can lead to behaviours like buying merchandise or publicly defending the figure. However, strong emotional ties also heighten vulnerability; when a media figure fails morally, fans may experience emotional distress akin to a breakup (He & Sun, 2022).

Other affective features include identifying as a fan—which in itself shapes behaviour—and developing negative feelings toward anti-fans. Fans often see anti-fans as a threat to their identity, which can spark online conflict (Yin, 2021). These affective dynamics are particularly strong in PSI, fostering loyalty, emotional fulfilment, and intensified engagement. Emotional bonds not only deepen attachment but also influence consumer actions aimed at maintaining closeness with the figure.

#### 4.2.3. Behavioural

When audiences long for a closer connection with a media figure, that desire can trigger a range of fan behaviours. Tukachinsky and Stever (2019) listed six behavioural characteristics: self-soothing, joining fan clubs, collecting memorabilia, creating fan fiction, seeking actual contact with the figure, and changing oneself because of the figure (e.g., altering appearance). Many of the fan studies we reviewed also document behaviours like joining clubs, collecting memorabilia, writing fan fiction, or seeking direct contact with the figure. These can all be broadly understood as fan behaviours. Consequently, it can be inferred that when individuals identify as fans, they are likely to exhibit such behaviours. However, ‘changing oneself because of a celebrity’ may not be exclusive to fan groups.

Audiences often seek genuine interaction with media figures they admire, with fans being the most prominent group. The rapid advancement of the internet, particularly through social media, has considerably altered the relationship between audiences and media personalities, fostering a closer connection. SMI have reaped the most benefits from these changes, emerging as the type of media personality most visibly affected by this shift. Additionally, traditional media celebrities, such as singers and actors, are increasingly using social media to present themselves and enhance their communication and interaction with fans. A substantial body of literature has extensively addressed these developments.

### 4.3. Parasocial Attachment

Compared to PSI and PSRs, PSA remains significantly underexplored in the literature, with only one study explicitly addressing it in this review. This limited representation may not simply reflect a lack of empirical interest, but rather three underlying challenges. First, PSA is often lumped together with PSRs, since both involve emotional bonds. But PSA is different—it is about emotional dependency and a sense of psychological security, not just relational continuity. Second, without standardised measurement tools, it is hard to operationalise PSA empirically, so many studies fall back on PSR constructs. Third, research has tended to focus on observable interactions and relational dynamics, leaving the deeper, more internalised attachment processes relatively unexplored.

This gap points to a clear direction for future work. We need sharper conceptual definitions and dedicated measures to really capture how attachment operates in mediated contexts.

It is also worth thinking about how different media formats shape these processes. Serial television and streaming platforms, for instance, offer repeated exposure and narrative continuity—conditions that favour PSRs. Social media, and especially influencer cultures, encourage frequent, low-effort interactions that intensify PSI. Algorithmic recommendations, real-time engagement, and self-disclosure all amplify perceived intimacy, fundamentally reshaping the context in which parasocial experiences take root.

### 4.4. Parasocial Experience as Parasocial Contact

Beyond its role in shaping individual engagement, parasocial experience can also be conceptualised as a form of parasocial contact. Drawing on mediated intergroup contact theory (Schiappa et al., 2005), interactions with media figures—particularly those representing outgroups—can function as indirect forms of social contact.

A growing body of research suggests that such parasocial interactions can influence attitudes, reduce prejudice, and shape moral evaluations. For instance, exposure to media characters from marginalised groups has been shown to foster empathy and improve intergroup attitudes, even in the absence of direct interpersonal interaction (e.g., Bond, 2021; Bond & Miller, 2021).

Framing parasocial experience as parasocial contact gives it broader theoretical significance beyond individual engagement. It shows that PSI and PSRs are not just about perceived intimacy—they can also serve as pathways for media consumption to produce real, measurable changes in attitudes and social perceptions (Hoffner & Bond, 2022).

This perspective is particularly relevant in contemporary digital environments, where algorithmically curated content increases repeated exposure to specific media figures, thereby amplifying the potential impact of parasocial processes on social perception and behaviour.

### 4.5. Limitations of Research

This review has several limitations. First, by focusing on studies published between 2019 and 2024, we ensured relevance to recent digital media developments—but this period also overlaps with the COVID-19 pandemic. So our findings may reflect pandemic-shaped behaviours (e.g., increased livestreaming, virtual idol engagement, and isolation-driven parasocial interaction) more than stable, post-pandemic patterns. Also, we did not include earlier foundational studies, which may have narrowed our theoretical lens. And limiting ourselves to open-access articles could have introduced selection bias.

Second, the geographical concentration of studies in China and the United States introduces a cultural bias that may restrict the transferability of findings. Audiences’ interpretations of media figures and parasocial processes are deeply shaped by local cultural norms and media ecologies, which vary significantly across regions.

Third, although the review included diverse methodological approaches, most studies employed quantitative methods such as surveys. This methodological emphasis may limit understanding of the nuanced emotional and relational dynamics of PSE, which are often better explored through qualitative or mixed-methods research.

Finally, empirical engagement with PSA was minimal, with only one study addressing it explicitly. This reflects its status as an under-theorised and emerging construct, resulting in an incomplete synthesis of the deeper affective dimensions of PSE.

## 5. Conclusions and Directions for Future Research

PSE has long been marginalised in academic discourse, in part due to early assumptions that such experiences were symptomatic of psychological dysfunction or social withdrawal. This perception has historically constrained scholarly engagement, casting para-sociality as deviant rather than normative. However, in recent years, researchers have increasingly recognised the complexity and emotional depth of PSE, particularly as digital platforms have made media figures more accessible and emotionally available. This shift has enabled a more nuanced understanding of how parasocial phenomena shape contemporary media–audience relationships.

This review analysed 59 empirical studies published between 2019 and 2024 that specifically examined the characteristics of PSE among audiences. The findings reveal that PSI and PSRs are deeply embedded in everyday media use, influencing audience cognition, affect, and behaviour. Many studies characterise these relationships as resembling friendships, with some recent work even extending the metaphor to familial or caregiving bonds. These findings suggest that PSE is not peripheral but central to how individuals navigate the mediated social world, offering mechanisms of attachment, belonging, and emotional regulation across the lifespan.

Despite growing interest, certain aspects of PSE remain underexplored. In particular, PSA has received limited empirical attention, which may reflect not only its conceptual ambiguity and overlap with PSRs, but also the lack of dedicated measurement tools and a broader theoretical tendency to prioritise observable interactions over deeper attachment processes. Although PSA was introduced over a decade ago as a distinct construct within parasocial theory (Stever, 2013; Tukachinsky & Stever, 2019), only one study in this review explicitly addressed it. Given the theoretical links between PSA and attachment styles, future research should explore how individual differences in attachment orientation influence the intensity and stability of audience–media figure bonds.

There is also a need for greater methodological diversity. The reviewed literature is predominantly quantitative, relying on structured questionnaires and cross-sectional designs. To better understand the motivations, meanings, and contextual nuances of PSE, future studies would benefit from employing qualitative approaches, such as interviews, ethnography, or narrative analysis. These methods are particularly suited to uncovering the subjective dimensions of parasociality, especially in underrepresented populations such as LGBTQ audiences, adolescents, or marginalised fan communities.

Finally, this review highlights the behavioural implications of PSE, particularly in the domain of intimacy-driven consumption. Audiences often express loyalty and identification through symbolic actions, including purchase decisions, content sharing, and public endorsements. Future research could examine how parasocial bonds influence consumer behaviour in emotional rather than rational terms. Such work would offer valuable insights for practitioners in marketing, platform design, and audience development, enabling more ethically responsive and emotionally attuned engagement strategies. This review makes three key contributions to the literature. First, it provides a systematic conceptual distinction between PSI, PSRs, and PSA, clarifying their differences in temporal, emotional, and behavioural dimensions. Second, it identifies parasocial attachment as a critically underexplored construct, highlighting the need for greater theoretical and empirical attention. Third, it advances the understanding of parasocial experience as a multi-layered process that not only shapes individual engagement but also has broader implications for media consumption and social behaviour. Importantly, these findings suggest that PSE should not be understood solely as a form of individual emotional engagement, but also as a mechanism with broader implications for attitude formation, identity construction, and social behaviour.

## Figures and Tables

**Table 1:** PSI scale.

Response	Process	Item Example
Cognitive	Attention allocation	I carefully followed the behaviour of PERSONA.
	Comprehension of persona’s action and situation	I hardly thought about why PERSONA did certain things. s/he didi. (inverted)
	Activation of prior media and life experience	I kept wondering if I knew persons that are similar to PERSONA.
	Evaluations of persona and life experience	I became aware of aspects of PERSONA that I really liked or disliked.
	Anticipatory observation	I kept asking myself how things would evolve around PERSONA.
	Construction of relations between persona and self	Occasionally, I wondered if PERSONA was similar to me or not.
Affective	Sympathy/antipathy	Sometimes I really loved PERSONA for what s/he did.
	Empathy/counter empathy	If PERSONA felt bad, I felt bad (Inverted).
	Emotion/counter empathy	PERSONA left me rather sober and unaffected. (inverted)
Behavioural	Nonverbal behaviour (e.g., mimics, gestures)	Occasionally, I said something to PERSONA on impulse.
	Behavioural intentions	Sometimes I felt like speaking out on PERSONA.

Source: (Schramm & Hartmann, 2008).

**Table 2:** EPSI scale.

**While watching the clip, I had the feeling that [name].**
1. was aware of me. 2. knew I was there. 3. knew I was aware of him/her. 4. knew I paid attention to him/her. 5. knew that I reacted to him/her. 6. reacted to what I said or did.

Source: (Hartmann & Goldhoorn, 2011).

**Table 3:** Summary information from the selected articles.

Tittle	Authors, Year	Location	Study Design & Sample	Instrument	Type of Relationship	Category
YouTube celebrities and parasocial interaction: Using feedback channels in mediatized relationships	Rihl and Wegener (2019)	Europe	Quantitative *n* = 174	Online survey	YouTube user and SMI	PSR -Compensation needs -SMIs make friends with their followers
Parasocial forgiveness: The roles of parasocial closeness and offence perceptions	Osterman and Hecmanczuk (2020)	USA	Quantitative *n* = 185	Online survey	Participants and actor	PSR -Personality type (dreamers) -Friendship makes it positive for fans to forgive celebrities who make mistakes
Reputation matters: parasocial attachment, narrative engagement, and the 2018 Taylor Swift political endorsement	Nisbett and Schartel Dunn (2021)	USA	Quantitative *n* = 153	Online survey	Undergraduate students and singer	PSA -
Microblogging reactions to celebrity endorsement: effects of parasocial relationship and source factors	W. Gong and Li (2019)	China	Quantitative *n* = 862	Questionnaire	Weibo users and actor	PSR -Compensation needs -Feel actor likes friend
Tweens’ Wishful Identification and Parasocial Relationships with YouTubers	Tolbert and Drogos (2019)	USA	Quantitative *n* = 161	Survey	Tweens and SMI	PSR -Compensation needs -Tweens’ attachment to celebrities
‘There’s a Starman waiting in the sky’: Mourning David #Bowie on Twitter	Van den Bulck and Larsson (2019)	/	Mixed *n* = 252,318	Content analysis and thematic analysis	Twitter users and singer	PSR -Compensation needs -Fans mourn a celebrity’s death like losing a relative
The Impact of Moral Expectancy Violations on Audiences’ Parasocial Relationships with Movie Heroes and Villains	Bonus et al. (2021)	USA	Quantitative *n* = 441	Online survey	Participants and movie character	PSR -Compensation needs -The audience cares about the character’s sense of morality
The Influence of Female Lead Characters in Political TV Shows: Links to Political Engagement	Hoewe and Sherrill (2019)	Malaysia	Quantitative *n* = 28	Online survey	Fans and television character	PSR -Compensation needs -Characters make friends with their audiences
Antecedents of Microblogging Users’ Purchase Intention Toward Celebrities’ Merchandise: Perspectives of Virtual Community and Fan Economy	A. Yang and Shim (2020)	China	Quantitative *n* = 297	Online survey	Fans and /	PSR -Personality type -Purchases reflect long-term fan–celebrity relationships
“They’re Worth My Investment”: Cultivating Intimacy through Fan-lead Financial and Support Initiatives among BTS Fans	Donabedian (2021)	Korean	Qualitative *n* = 22	Semi-structured interview	Fans and singer	PSR -Personality type -Purchases reflect long-term fan–celebrity relationships
The moderating role of parasocial relationships in the associations between celebrity endorser’s credibility and emotion-based responses	Burnasheva and Suh (2022)	Korean	Quantitative *n* = 299	Online survey	Millennials fans and singer	PSR -Personality type -Fans purchase intention related to celebritiy
Escapism and motivation: Understanding K-pop fans well-being and identity	Jenol and Pazil (2020)	Malaysia	Qualitative *n* = 8	Semi-structured interview	Fans and singer	PSR -Compensation needs -Fans use their relationship with idols
How does a celebrity make fans happy? Interaction between celebrities and fans in the social media context	M. Kim and Kim (2020)	USA	Quantitative *n* = 642	Questionnaire	Fans and /	PSI (Affective) -Feeling of intimacy -Defining self as fan -Purchase intention
Does parasocial interaction with weight loss vloggers affect compliance? The role of vlogger characteristics, consumer readiness, and health consciousness	Sakib et al. (2020)	USA	Quantitative *n* = 378	Online survey	Undergraduate students and SMI	PSI (Behavioural) -Change self because of figure -Alter appearance
The Development and Influence of Parasocial Relationships with Television Characters: A Longitudinal Experimental Test of Prejudice Reduction Through Parasocial Contact	Bond (2021)	USA	Quantitative *n* = 146	Questionnaire	Undergraduate students and television character	PSR -Compensation needs -Outgroup characters ease viewers’ emotional gaps and biases through media exposure
An emergent algorithmic culture: The data-ization of online fandom in China	Yin (2020)	China	Qualitative *n* = 30	Semi-structured interviews	Fans and /	PSI (Affective) -Feeling of intimacy -Defining self as fan -Purchase intention
How Social Media Influencers Foster Relationships with Followers: The Roles of Source Credibility and Fairness in Parasocial Relationship and Product Interest	Yuan and Lou (2020)	/	Quantitative *n* = 355	Online survey	Followers and SMI	PSR -Personality type -Consumers may trust influencers as much as friends
Parasocial Relationships with President Trump as a Predictor of COVID-19 Information Seeking	Kelly et al. (2020)	USA	Quantitative *n* = 529	Online questionnaire	Participants and politicians	PSR -Compensation needs -The audience alleviates discomfort by identifying with a character or brand
The effect of celebrity endorsement on destination brand love: A comparison of previous visitors and potential tourists	H. Zhang et al. (2020)	China	Quantitative *n* = 1044	Questionnaire	Consumers and actor	PSI (Behavioural) -Change self because of figure -Travel destination
The Politics of Being “Cait”: Caitlyn Jenner, Transphobia, and Parasocial Contact Effects on Transgender-Related Political Attitudes	Miller et al. (2020)	USA	Quantitative *n* = 1940	Online survey	American adults and reality show star	PSI (Affective) -The character makes one feel better
“Dude I’ve Never Felt This Way Towards a Celebrity Death”: Parasocial Grieving and the Collective Mourning of Kobe Bryant on Reddit	Bingaman (2022)	/	Quantitative *n* = 268	Content analysis	Riddit users and athletes	PSR -Personality type -Fans feel the same grief for celebrities as they do for friends
Engagement With the Gurus of Gaming Culture: Parasocial Relationships to Let’s Play	Kreissl et al. (2021)	Europe	Quantitative *n* = 859	Online survey	Fans and SMI	PSR -Personality type -Fans emotional connection and cultural identity with players
My baby should feel no wronged!”: Digital fandoms and emotional capitalism in China	Yin (2021)	China	Qualitative *n* = 20	Participated observation and Semi-structured interview	ACG Fans and /	PSI (Affective) -Feeling of intimacy -Defining self as fan -Purchase intention
“Stop the unattainable ideal for an ordinary me!” fostering parasocial relationships with social media influencers: The role of self-discrepancy	Aw and Chuah (2021)	/	Quantitative *n* = 361	Online survey	Fans and SMI	PSR -Compensation needs -Effect fans purchase intention
From parasocial to parakin: Co-creating idols on social media	Yan and Yang (2021)	China	Qualitative *n* = 36	In depth interview	Fans and idol	PSR -Personality type -Fans’ emotional needs and identification with their idols
Social TV viewers’ symbolic parasocial interactions with media characters: A topic modelling analysis of viewers’ comments	J. Kim and Sintas (2021)	/	Quantitative *n* = 214,632	Content analysis	Viewers and television character	PSI (Cognitive) -Sense of knowing the figure well
Friend or just fans? Parasocial relationships in online television fiction communities	Lacalle et al. (2021)	Europe	Mixed *n* = 100	Content analysis	Viewers and television character	PSR -Personality type -Participants’ emotional connection with and identification of the character
The impact of Internet celebrity characteristics on followers’ impulse purchase behavior: the mediation of attachment and parasocial interaction	Chen et al. (2021)	China	Quantitative *n* = 500	Online survey	Follower and SMI	PSI (Cognitive) -Sense of knowing the figure well -Impulse purchase behaviour
Beyond fans: The relational labor and communication practices of creators on Patreon	Bonifacio et al. (2023)	USA	Qualitative *n* = 21	Semi-structured interviews	Creators and SMI	PSR -Personality type -Sponsors see creators as friends rather than as commodities
Digital ventriloquism and celebrity access: Cameo and the emergence of paid puppeteering on digital platforms	Drenten and Psarras (2023)	USA	Qualitative *n* = 69	Content analysis	Fans and Reality stars	PSR -Compensation needs -Effect fans purchase intention
YouTube as my space: The relationships between YouTube, social connectedness, and (collective) self-esteem among LGBTQ individuals	Bond and Miller (2021)	USA	Quantitative *n* = 482	Online questionnaire	LGBTQ and SMI	PSR -Compensation needs -LGBT individuals use social media to meet their need for emotional and social support
‘I’m like you, and I like what you like’ sustainable food purchase influenced by vloggers: A moderated serial-mediation model	Z. Xu et al. (2021)	China	Quantitative *n* = 382	Questionnaire	TikTok user and SMI	PSI (Behavioural) -Change self because of figure -Purchase intention
You follow fitness influencers on YouTube. But do you actually exercise? How parasocial relationships, and watching fitness influencers, relate to intentions to exercise	Sokolova and Perez (2021)	Europe	Quantitative *n* = 300	Questionnaire	Millennial generation and SMI	PSR -Compensation needs -Compensate for behavioural or emotional deficiencies
An Attachment Perspective on Favorite Media Figures	MacNeill and DiTommaso (2022)	Canada and USA	Quantitative *n* = 200	Online survey	Audiences and television character	PSR -Personality type -Individual’s attachment style and the characteristics of the media figure they choose
Breaking up with my idol: A qualitative study of the psychological adaptation process of renouncing fanship	He and Sun (2022)	China	Qualitative *n* = 8	In depth interview	Fans and idol	PSR -Compensation needs -Fans’ psychological adjustment process to make up for the emotional loss PSI (Cognitive) -Less critical in the event of a scandal/transgression
Parasocial relations and social media influencers’ persuasive power. Exploring the moderating role of product involvement	Balaban et al. (2022)	Europe	Quantitative *n* = 190	Online survey	Female users and SMI	PSR -Personality type -The psychological process of forming relationships with influencers on social media
Celebrity brand break-up: Fan experiences of para-loveshock	Jones et al. (2022)	USA	Qualitative *n* = 15	In depth interview	Fans and actor	PSR -Personality type -The emotional processes and practices that fans engage in following a perceived loss
What drives Taobao live streaming commerce? The role of parasocial relationships, congruence and source credibility in Chinese consumers’ purchase intentions	Rungruangjit (2022)	Thailand	Quantitative *n* = 454	Questionnaire	Consumers and SMI	PSR -Compensation needs -Effect fans purchase behaviours
The effect of hotel livestreaming on viewers’ purchase intention: Exploring the role of parasocial interaction and emotional engagement	Shen et al. (2022)	China	Quantitative *n* = 348	Questionnaire	Audiences and SMI	PSI (Affective) -Feeling of intimacy
From interaction to relationship: Rethinking parasocial phenomena in travel live streaming	Deng et al. (2022)	China	Qualitative *n* = 18	Netnography and interviews	Users and SMI	PSR -Personality type -Viewers end to rely on emotional resonance and personal relationship building PSI (Cognitive) -Sense of knowing the figure well
“Leave Britney alone!”: parasocial relationships and empathy	Scherer et al. (2022)	USA	Quantitative *n* = 122	Online survey	Fans and singer	PSR -Personality type -Individuals’ empathy and perspectives.
Behind the lab coat: How scientists’ self-disclosure on Twitter influences source perceptions, tweet engagement, and scientific attitudes through social presence	A. L. Zhang and Lu (2022)	USA	Quantitative *n* = 1458	Online questionnaire	Participants and SMI	PSR -Compensation needs -Various self-presentation methods are used to balance public perceptions. PSI (Cognitive) -Sense of knowing the figure well
The study on the impact of short video tourism Vloggers at social media platform on online sharing intention	Zhao et al. (2022)	China	Quantitative *n* = 588	Questionnaire	Tourists and SMI	PSR -Compensation needs -People interact with fitness bloggers to compensate for behavioural or emotional deficiencies.
How nostalgic taste on the screen stimulates the consumption of time-honoured restaurants: The mediation role of parasocial interaction	J. Yang et al. (2022)	China	Quantitative	Online survey	Consumers and SMI	PSI (Affective) -Feeling of intimacy -Purchase intention
How Do Comeback Korean Pop Performers Acquire Audience Empathetic Attachment and Sustained Loyalty? Parasocial Interactions Through Live Stream Shows	Ma et al. (2022)	China and Korea	Quantitative *n* = 288	Questionnaire	K-pop fans (China and Korean) and Singer	PSI (Affective) -Feeling of intimacy -Purchase intention
Finding love in online games: Social interaction, parasocial phenomenon, and in-game purchase intention of female game players	A. D. Gong and Huang (2023)	China	Quantitative *n* = 615	Online questionnaire	Female game user and game character	PSI (Affective) -The character makes one feel better -Feeling of intimacy -Purchase intention
Presentation of celebrities’ private life through visual social media	Klostermann et al. (2023)	Europe	Quantitative *n* = 1437	Content analysis	Male European soccer players & /	PSR -Compensation needs -Celebrities compensate for the lack of professional content through private sharing
How and when social media influencers’ intimate self-disclosure fosters purchase intentions: the roles of congruency and parasocial relationships	Koay et al. (2023)	Malaysia	Quantitative *n* = 232	Questionnaire	Followers and SMI	PSR -Compensation needs -Psychological and emotional needs of consumers in purchase intention
Why viewers send paid gifts: The role of social influence on massively multiplayer online games live streaming	M. Gong et al. (2023)	China	Quantitative *n* = 988,829	Self-reported survey	Viewers and SMI	PSR -Compensation needs -Psychological or social needs by forming a bond with the host or game content
From social media diet to public riot? Engagement with “greenfluencers” and young social media users’ environmental activism	Knupfer et al. (2023)	Europe	Quantitative *n* = 865	Questionnaire	Young Germans and socialist	PSR -Compensation needs -People make up for their lack of action on the environment
Para-kin relationship between fans and idols: a qualitative analysis of fans’ motivations for purchasing idol-dolls	Z. Zhang (2023)	China	Qualitative *n* = 13	In depth interview	Gen Z fans and idol	PSR -Compensation needs -Individuals compensate for emotional deficiencies or deficits by interacting with idol dolls -Purchase behaviour
Downloading appetite? Investigating the role of parasocial relationship with favorite social media food influencer in followers’ disordered eating behaviors	Shabahang et al. (2024)	Iran	Quantitative *n* = 405	Online survey	Users and SMI	PSR -Compensation needs -Admiration for celebrities can influence unhealthy eating habits
Tourist acceptance of ChatGPT in travel services: the mediating role of parasocial interaction	H. Xu et al. (2024)	China	Quantitative *n* = 331	Online questionnaire	ChatGPT user and AI chatbot ChatGPT	PSI (Cognitive) -Sense of knowing the figure well
When TV neighbours become good friends: Understanding Neighbours fans’ feelings of grief and loss at the end of the series	Gerace (2024)	Australia	Quantitative *n* = 1289	Online questionnaire	Fans and Television Characters	PSR -Personality type -Fans feelings of closeness, similarity, and empathy toward favourite characters
“It’s All Just F*cking Impossible:” The influence of Taylor Swift on fans’ body image, disordered eating, and rejection of diet culture	Pope and Rose (2024)	USA	Qualitative *n* = 200	Content analysis	Fans and singer	PSR -Compensation needs -Personal experience disclosure inspires fans
“It’s between me and myself”: Inverse parasocial relationships in addressing (imagined) podcast listeners	Sharon and John (2024)	Israel	Qualitative *n* = 12	In depth interview	Audience and SMI	PSR -Personality type -Audiences feelings of closeness
Parasocial Interactions of Indonesian Beauty Vloggers in the Digital Age: Do they Impact Purchases by Millennial Netizens?	Kuswati et al. (2024)	Indonesia	Quantitative *n* = 450	Online questionnaire	Millennial fans and SMI	PSI (Affective) -Feeling of intimacy -Purchase intention
Parasocial interactions and parasocial relationships on Instagram: An in-depth analysis of fashion and beauty influencers	Zhou et al. (2024)	Japan	Quantitative *n* = 215	Regression analysis	Followers and SMI	PSI (Cognitive) -Sense of knowing the figure well -Replying to comments, strong PSR
How travel vlog audience members become tourists: Exploring audience involvement and travel intention	Hu et al. (2024)	China	Quantitative *n* = 454	Online questionnaire	Audience and SMI	PSI (Affective) -Feeling of intimacy

## Data Availability

No new data were created or analysed in this study.
